# Fungal Extracellular Vesicles Are Involved in Intraspecies Intracellular Communication

**DOI:** 10.1128/mbio.03272-21

**Published:** 2022-01-11

**Authors:** Tamires A. Bitencourt, Otavio Hatanaka, Andre M. Pessoni, Mateus S. Freitas, Gabriel Trentin, Patrick Santos, Antonio Rossi, Nilce M. Martinez-Rossi, Lysangela L. Alves, Arturo Casadevall, Marcio L. Rodrigues, Fausto Almeida

**Affiliations:** a Department of Biochemistry and Immunology, Ribeirao Preto Medical School, University of São Paulo, Ribeirao Preto, São Paulo, Brazil; b Department of Genetics, Ribeirão Preto Medical School, University of São Paulo, Ribeirão Preto, São Paulo, Brazil; c Gene Expression Regulation Laboratory, Carlos Chagas Institute, Fiocruz, Curitiba, PR, Brazil; d Department of Molecular Microbiology and Immunology, Johns Hopkins Bloomberg School of Public Health, Baltimore, Maryland, USA; e Instituto de Microbiologia Paulo de Góes, Universidade Federal do Rio de Janeiro, Rio de Janeiro, Brazil; Lundquist Institute for Biomedical innovation at Harbor-UCLA Medical Center; Yonsei University

**Keywords:** fungal infections, extracellular vesicles, fungal biology, *Aspergillus fumigatus*, *Candida albicans*, cellular communication, *Paracoccidioides brasiliensis*

## Abstract

Fungal infections are associated with high mortality rates in humans. The risk of fungal diseases creates the urgent need to broaden the knowledge base regarding their pathophysiology. In this sense, the role of extracellular vesicles (EVs) has been described to convey biological information and participate in the fungus-host interaction process. We hypothesized that fungal EVs work as an additional element in the communication routes regulating fungal responses in intraspecies interaction systems. In this respect, the aim of this study was to address the gene regulation profiles prompted by fungal EVs in intraspecies recipient cells. Our data demonstrated the intraspecies uptake of EVs in pathogenic fungi, such as Candida albicans, Aspergillus fumigatus, and Paracoccidioides brasiliensis, and the effects triggered by EVs in fungal cells. In C. albicans, we evaluated the involvement of EVs in the yeast-to-hypha transition, while in *P. brasiliensis* and A. fumigatus the function of EVs as stress transducers was investigated. *P. brasiliensis* and A. fumigatus were exposed to an inhibitor of glycosylation or UV light, respectively. The results demonstrated the role of EVs in regulating the expression of target genes and triggering phenotypic changes. The EVs treatment induced cellular proliferation and boosted the yeast to hyphal transition in C. albicans, while they enhanced stress responsiveness in A. fumigatus and *P. brasiliensis*, establishing a role for EVs in fungal intraspecies communication. Thus, EVs regulate fungal behavior, acting as potent message effectors, and understanding their effects and mechanism(s) of action could be exploited in antifungal therapies.

## INTRODUCTION

Fungal infections are responsible for over 1.6 million deaths per year. It is estimated that more than a billion cases of severe fungal diseases affect the world population. Despite these numbers it is likely that they represent an underestimate of the fungal diseases that ail humans (1, 2). The diseases caused by Aspergillus spp., *Candida* spp., and the agents of mycoses such as *Paracoccidioides* species are among the deadliest mycoses (1, 2).

In recent years, extracellular vesicles (EVs) have been studied in cell-walled microorganisms. In fungi, they were first described in 2007 in Cryptococcus neoformans (3). So far, EVs have been characterized in approximately 20 fungal species, including yeasts forms of H. capsulatum, *Sporothrix schenckii*, C. parapsilosis, Saccharomyces cerevisiae, *Malassezia sympodialis*, *P. brasiliensis*, C. albicans, *Pichia fermentans*, C. gattii, *S. brasiliensis*, *P. lutzii*, and *Exophiala dermatitidis* (4–13) and filamentous fungi such as *Alternaria infectoria*, Trichophyton interdigitale, *Rhizopus delemar*, Fusarium oxysporum f. sp. *vasinfectum*, Trichoderma reesei, Aspergillus fumigatus, and Aspergillus flavus (14–20).

EVs function as vehicles carrying complex cargoes with diverse biological functions, including proteins, carbohydrates, pigments, nucleic acids, and lipids. EVs can contribute to fungal infection outcomes (21). EVs likely have roles in bidirectional communication, raising the possibility of communication between fungal cells (22). Previous reports have demonstrated the participation of fungal EVs in biofilm formation (10), stimulation of cytokine production (6, 9, 11, 15, 20, 23), and favoring pathogen infection (4, 24). Bidirectional communication mediated by fungal EVs has been demonstrated in the interaction of fungal cells with plants (25, 26) and in communication with mammalian cells (27, 28).

The possibility of EV-mediated virulence transfer and/or antifungal resistance between strains has gathered attention. In the Cryptococcus model, previous studies demonstrated that the Vancouver Island outbreak strain of *C. deuterogatti*, namely, R265, can transfer its ability to proliferate within the host macrophages to an avirulent strain, which was attributed to an EV-regulated process (11, 29). Another report showed that the supernatant from a highly virulent strain of C. neoformans culture stimulated the pathogenic potential of a less virulent isolate, an effect also attributed to EVs (30). Furthermore, EVs isolated from biofilms of wild-type (WT) C. albicans restored the biofilm production and fluconazole tolerance in mutant strains with a negative background in orthologs of endosomal sorting complexes required for transport (ESCRT) subunits (31). Recently, a study advocated the role of EVs from C. albicans as messaging compartments involved in growth, morphogenesis, and biofilm production (32). Altogether, these studies suggested a role for fungal EVs in intraspecies communication; however, at the molecular level, many aspects related to the mechanisms switched on by EVs remain to be investigated. Here, we sought to analyze the fungal cellular communication mediated by EVs using the fungal pathogens *P. brasiliensis*, A. fumigatus, and C. albicans using multiple approaches. Our data demonstrate that fungal EVs mediate cellular communication by regulating the expression of target genes and by controlling cellular proliferation.

## RESULTS

### Intercellular transfer of fungal EVs.

**FIG 1:**
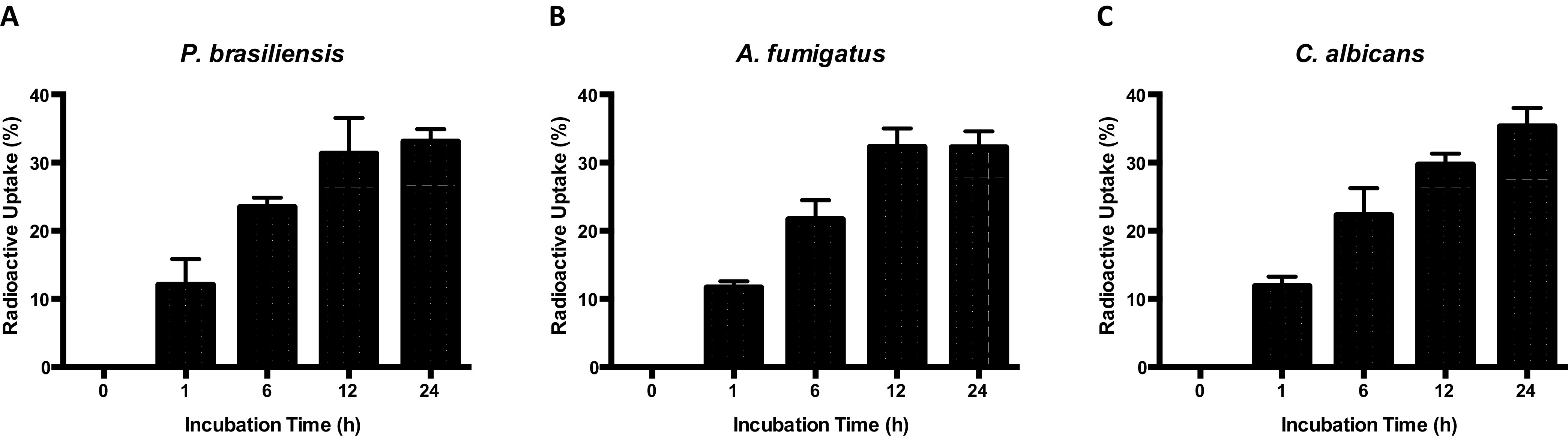
Evaluation of extracellular vesicles (EVs) uptake in different fungal species. The absorption of radioactive VEs was evaluated after 0, 1, 6, 12, and 24 h in the following yeast cells: *P. brasiliensis* (A), A. fumigatus (B), and C. albicans (C).

Because EVs can transport multiple molecules that play essential roles in fungal biology (22, 28, 33), we examined whether fungal EVs would be transferred intercellularly from cells of the same species. Using [1-^14^C] palmitic acid metabolic labeling, radiolabeled fungal EVs were produced as previously described (34, 35). Radioactive assays confirmed that fungal EVs produced by *P. brasiliensis* (Fig. 1A), A. fumigatus (Fig. 1B), and C. albicans (Fig. 1C) could be transferred from cell to cell within the same species. We tracked the radioactivity added to the fungal cell cultures during the time course after 0 h (control), 1 h, 6 h, 12 h, and 24 h from the addition of radiolabeled EVs to the cultures (Fig. 1). The fungal cells were pelleted and washed with phosphate-buffered saline (PBS), and pulse-chase measurements were performed. We verified that purified radiolabeled EVs were taken up by fungal cells or at least associated with the cell surface as the radioactive signal increased over time. In this respect, we posit that EVs are taken up and internalized, or at least associated, with the fungal cell surface.

### *P. brasiliensis* EVs as cellular communicator during ER stress.

**FIG 2:**
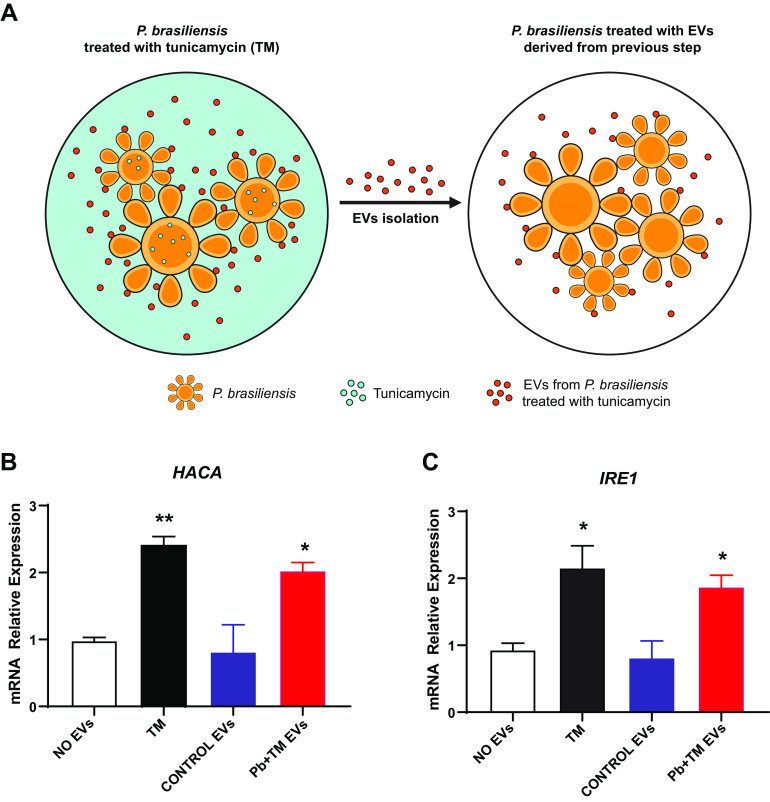
EVs from *P. brasiliensis* as cellular communicator during ER stress. (A) Schematic representation of EVs obtained after tunicamycin exposure. The relative expression of UPR genes *HACA* (B) and *IRE1* (C) was determined after EVs uptake (4 × 10^9^/ml EVs in 10^5^/ml recipient cells). Significantly different values are indicated by asterisks as determined using ANOVA followed by Tukey’s *post hoc* test (*P* < 0.05). The calibrator was the NO EVs condition, and the positive control was tunicamycin (TM).

We previously demonstrated that the genes *HACA* and *IRE1* showed increased expression during tunicamycin (TM) treatment (36), suggesting that they are involved in the endoplasmic reticulum (ER) stress response of *P. brasiliensis*. Thus, we purified EVs from *P. brasiliensis* treated with TM (TM EVs) and added these EVs to *P. brasiliensis* yeast cells that did not receive TM treatment (Fig. 2A). We observed that TM EVs increased *HACA* and *IRE1* expression significantly (Fig. 2B and C, red bars). On the other hand, fungal EVs obtained from untreated *P. brasiliensis* yeast cells (CONTROL EVs) were added to *P. brasiliensis* yeast cells, which did not alter *HACA* and *IRE1* expression (Fig. 2B and C, blue bars). PBS (NO EVs) was used as a negative control (white bar), while TM was the positive-control condition (black bars). These results strongly suggested that *P. brasiliensis* EVs could participate in intercellular communication during ER stress, possibly promoting fungal adaptive responses.

### EVs from A. fumigatus act as stress message effectors.

To verify whether the observations made with *P. brasiliensis* applied to other fungi, we isolated EVs from UV-irradiated cultures and from nonirradiated A. fumigatus cells (control). The EVs obtained from UV-treated cells were named UV EVs, whereas the EVs obtained from regular cultures from A. fumigatus were named CONTROL EVs. Our data showed an overall population size in the range of 100 to 200 nm and a minor population of EVs with sizes varying from 320 to 394 nm in regular cultures or a range of 240 to 614 nm in cultures that underwent UV irradiation (Fig. S1).

10.1128/mbio.03272-21.1 FIG S1 Histograms from particle-size distribution of extracellular vesicles (EVs) from C. albicans and A. fumigatus for each assessed condition. Download FIG S1, EPS file, 1.6 MB. Copyright © 2022 Bitencourt et al. 2022 Bitencourt et al. https://creativecommons.org/licenses/by/4.0/ This content is distributed under the terms of the Creative Commons Attribution 4.0 International license.

**FIG 3:**
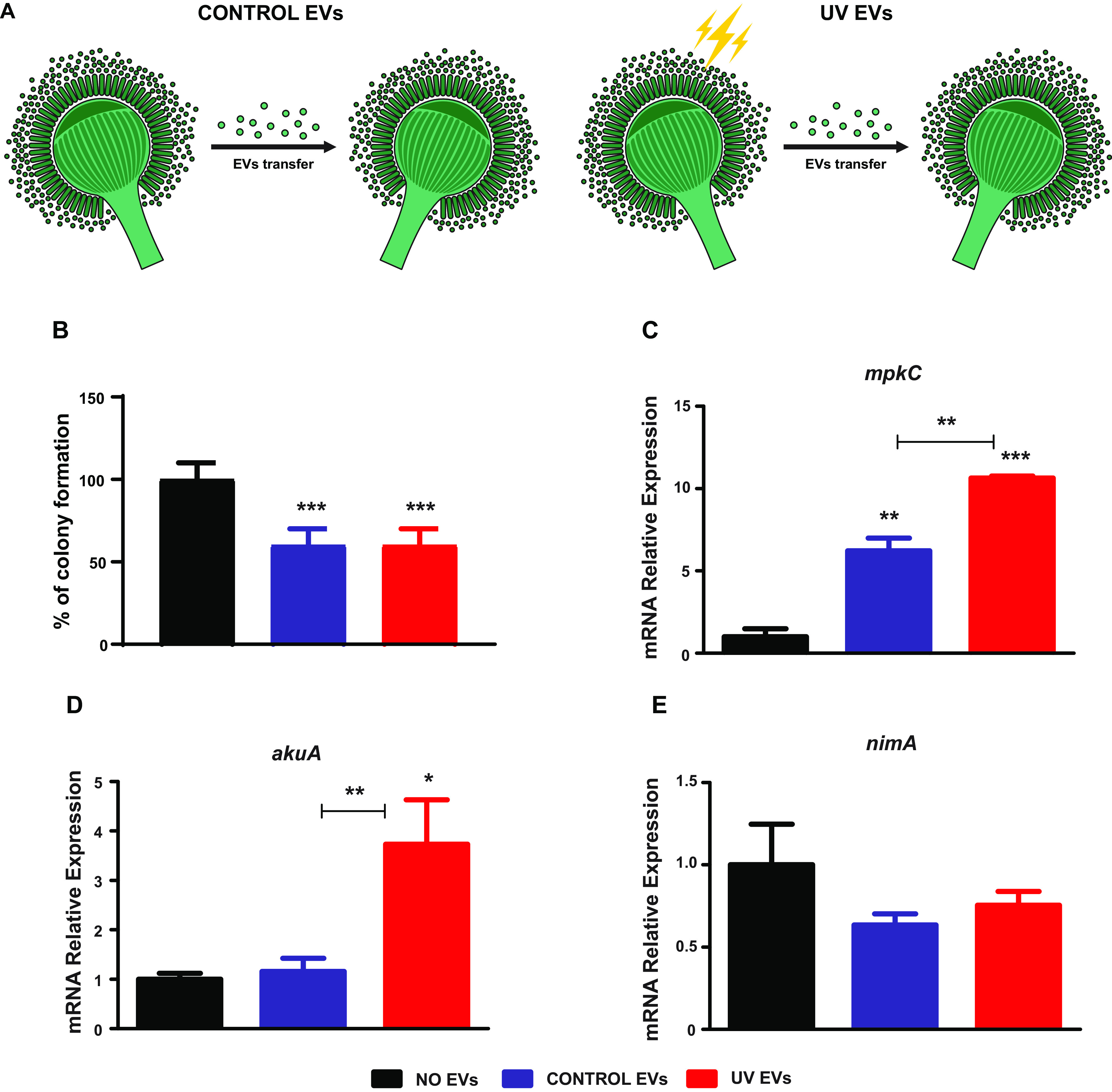
EVs from A. fumigatus as stress message effectors. (A) Schematic representation of EVs obtained from regular cultures, without UV exposure (CONTROL EVs), and EVs obtained from UV-exposed cultures (UV EVs). The thunder diagram represents UV light exposure. The EVs uptake was performed with 5 × 10^8^/ml of EVs in 10^4^/ml recipient cells. (B) The colony formation was counted and a percentage value was determined after EVs uptake. (C to E) RT-qPCR for A. fumigatus genes *mpkC* (C), *akuA* (D), and *nimA* (E). Relative expression was assessed using the NO EVs condition as a reference sample and 18S and *βtub* as a reference for normalization. Significantly different values are indicated by asterisks as determined using ANOVA followed by Tukey’s *post hoc* test (*, *P* < 0.05; **, *P* < 0.01; ***, *P* < 0.001).

EVs obtained from both A. fumigatus cultures after UV irradiation (UV EVs) or without UV irradiation (CONTROL EVs) (Fig. 3A) caused a prominent decrease in colony formation in A. fumigatus (Fig. 3B). Approximately 40% of colony reduction was achieved after EVs uptake (Fig. 3B). Thereafter, the gene expression analysis reinforced a possible role of these EVs as stress message effectors, showing the upregulation of the *mpkC* gene under both EVs uptake conditions. The *mpkC* gene, which encodes the mitogen-activated protein kinase (MAPK) ortholog, known as Hog1p, plays a role in adaptive responses to different stress agents, such as osmotic stress, oxidative stress, heat shock, and cell wall damage and also acts in cellular division regulation, as demonstrated in A. fumigatus (37, 38). The cultures that received UV EVs showed higher induction of the *mpkC* gene (Fig. 3C). In addition, UV EVs also caused a significant increase in the *akuA* transcript levels (Fig. 3D), supporting a role of EVs in cellular communication events. The *akuA* gene encodes the Ku70 component involved in DNA repair (39). In addition, the presence of EVs caused a subtle downregulation of the *nimA* gene, the encoding gene of cell cycle-regulated protein kinase, which may reflect another level of cell cycle regulation (Fig. 3E). To prove that the communication process relies only on intact EVs, we also tested heated EVs. In such cases, we evaluated the effects triggered by heated EVs from both conditions (CONTROL EVs and UV EVs) on colony formation and *akuA* modulation (Fig. S2). Similar profiles of colony formation were observed when recipient cells were treated with heated EVs or non-heated EVs (about 40 to 45% reduction). Conversely, the uptake of heated UV EVs did not impact *akuA* modulation. The condition control EVs (heated) were associated with a decrease in *akuA* transcript levels.

10.1128/mbio.03272-21.2 FIG S2 Analysis of the effects triggered by heated EVs in A. fumigatus. (A) RT-qPCR to assess the *akuA* transcript levels. Relative expression was assessed using the NO EVs condition as a reference sample and 18S and *βtub* as the reference of normalization. (B) The colony formation was counted, and a percent value was determined after heated EV uptake compared with the NO EVs condition. Significantly different values are indicated by asterisks as determined using ANOVA followed by Tukey’s *post hoc* test (*, *P* < 0.05; **, *P* < 0.01). Download FIG S2, EPS file, 0.9 MB. Copyright © 2022 Bitencourt et al. 2022 Bitencourt et al. https://creativecommons.org/licenses/by/4.0/ This content is distributed under the terms of the Creative Commons Attribution 4.0 International license.

### EVs induce hypha formation and regulate the cell cycle in C. albicans.

Next, we investigated whether EVs obtained from yeast culture and a yeast-to-hypha culture in the C. albicans model would mediate communication, affecting its morphological transition. Therefore, we isolated EVs from C. albicans from hypha-inducing cultures and from yeast cultures. We analyzed the size and distribution of EVs in both cultures (see Fig. S1 in the supplemental material). EVs obtained after C. albicans grown on cultures in YPD at pH 6.3 at 30°C were named CONTROL EVs, whereas EVs obtained from C. albicans grown on cultures in YPD at pH 7.4 at 37°C were named TRANS EVs. Our results showed a heterogeneous EV distribution profile within these conditions, and the majority of EVs obtained from yeast-to-hypha transition cultures (TRANS EVs) had a size range from 99 to 182 nm and minor populations with sizes varying from 23 to 85 nm and 232 to 440 nm. For EVs isolated from C. albicans yeast cultures (CONTROL EVs), the majority of EVs had a size range from 107 to 154 nm and a minor population in the range of 211 to 440 nm. Previous studies have demonstrated the heterogeneity of EVs from *Candida* spp., showing a distribution profile with population sizes ranging from 60 to 280 nm, and in C. albicans, the sizes varied from 100 nm to 600 nm (5, 40, 41).

**FIG 4:**
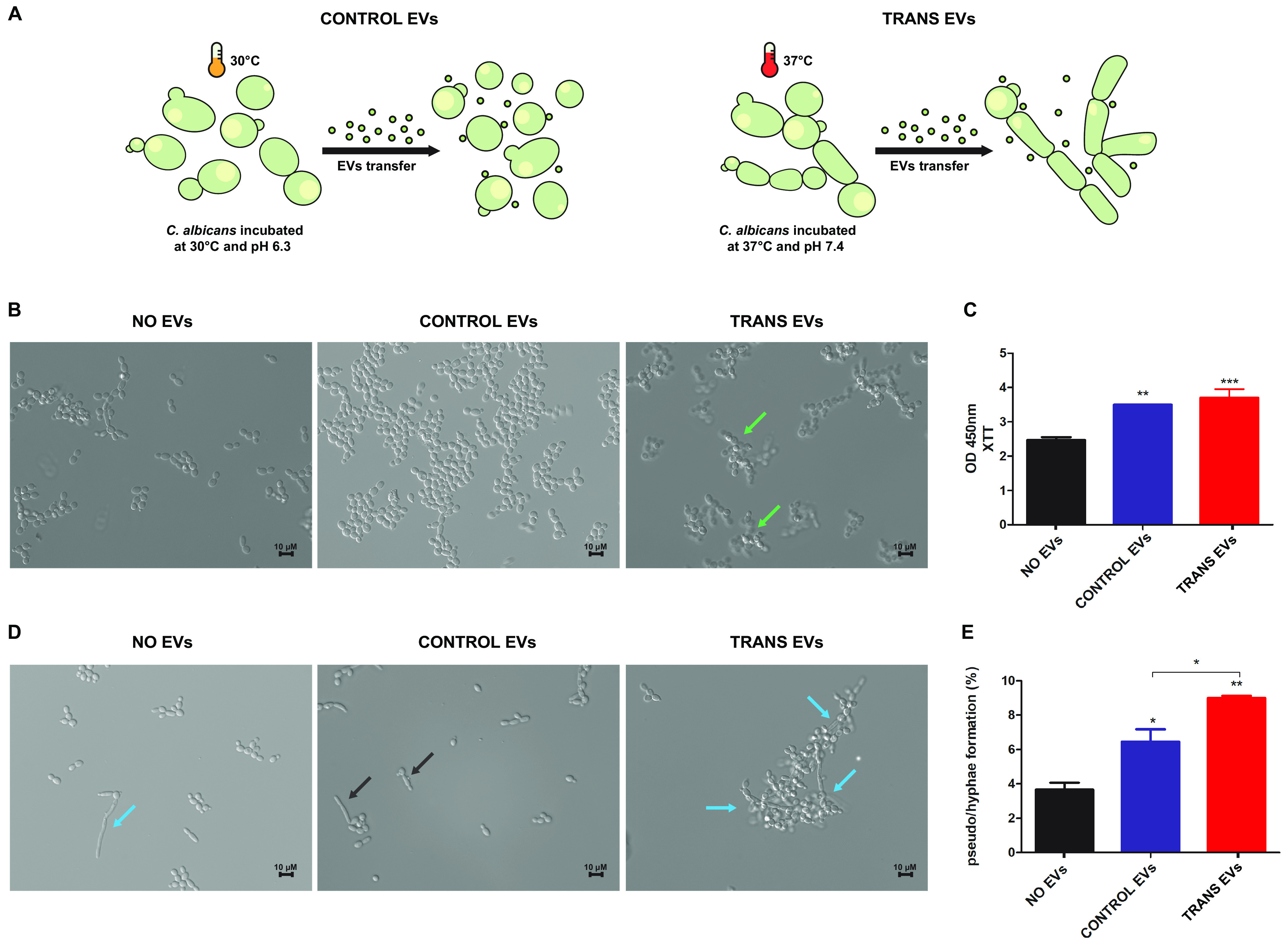
EVs heighten the cellular proliferation and pseudohypha formation in C. albicans. (A) Schematic representation of EVs obtained from yeast cultures (CONTROL EVs) and yeast-to-hypha cultures (TRANS EVs). (B) Morphological appearance was investigated by microscope images of C. albicans cells grown on YPD, pH 7.4, at 37°C after EVs uptake for 2 h. Green arrows depict clump structures observed in cultures that underwent TRANS EVs uptake. (C) XTT reduction assay to assess cellular proliferation after EVs uptake during 2 h of growth on transition condition. Identification of hypha and pseudohypha morphologies in C. albicans grown on transition condition for 4 h is shown. (D) Black arrows depict pseudohyphae and blue arrows depict hypha structures. (E) Estimation of morphotype abundance after 4 h of C. albicans growth on transition condition.

We also evaluated the gene expression profiles of selected genes (*HWP1*, *SAP5*, *CHT2*, and *SEC24*) during C. albicans growth in yeast-to-hypha transition cultures compared to yeast cultures after different incubation times, such as 0.5, 1, 2, and 4 h (Fig. S3). These genes were previously modulated in microarray data of C. albicans grown as hyphae with serum at 37°C, and *SAP5* (secreted aspartyl protease) and *HWP1* (cell wall protein hyphal wall protein 1) were highly upregulated (42). We identified a time-dependent modulation for the genes *CHT2* (endochitinase) and *SEC24* (ER to Golgi transport). A prominent upregulation was verified for the *HWP1* gene at all time points evaluated. The *SAP5* gene was upregulated at all times analyzed. Taking advantage of this information, we compared gene expression profiles after EVs uptake in C. albicans. Thus, the C. albicans cells were incubated with TRANS EVs or CONTROL EVs (Fig. 4A). Thereafter, the cells were recovered through centrifugation and resuspended in a yeast-to-hypha medium. By analyzing the morphological features of C. albicans under yeast-to-hypha conditions assessed throughout the incubation time points, we identified the occurrence of the three main morphologies presented by C. albicans, yeast, pseudohyphae, and hyphae. Moreover, the cultures that underwent TRANS EVs uptake appeared to present changes in yeast development in which a particular cellular clumping was detected at earlier time points (Fig. 4B). Our results also showed a possible increase in cellular proliferation after EVs uptake compared to NO EVs uptake cultures. The 2,3-bis-(2-methoxy-4-nitro-5-sulfophenyl)-5-[carbonyl (phenylamino)]-2H-tetrazolium hydroxide (XTT) assay reinforced the involvement of EVs in cell cycle regulation (Fig. 4C). Furthermore, during the course of growth, the cultures that received TRANS EVs displayed more developed hyphae than the other conditions, NO EVs and CONTROL EVs (Fig. 4D). The estimation of morphotype abundance also indicated that C. albicans cultures receiving EVs presented an enhancement of pseudohypha and/or hypha formation, which was mostly predominant after TRANS EVs treatment (Fig. 4E). In parallel, the effect of heated EVs on the regulation of morphotypes was analyzed. Although C. albicans treated with heated EVs displayed heightened pseudohypha and/or hypha formation (about 7%), regardless of the type of EV treatment, CONTROL EVs (heated) or TRANS EVs (heated) (Fig. S4), it was lower than uptake of intact TRANS EVs (about 9%).

10.1128/mbio.03272-21.3 FIG S3 Transition gene expression profile in C. albicans for selected genes such as *HWP1*, *SEC24*, *CHT2*, and *SAP5* after 0.5, 2, and 4 h. Control cultures were used as a reference for the modulation of transition cultures. The *RPP2B* and *TDH3* genes were used as normalizer genes. Significantly different values are indicated by asterisks, as determined using an unpaired *t* test (*, *P* < 0.05). Download FIG S3, EPS file, 1.7 MB. Copyright © 2022 Bitencourt et al. 2022 Bitencourt et al. https://creativecommons.org/licenses/by/4.0/ This content is distributed under the terms of the Creative Commons Attribution 4.0 International license.

10.1128/mbio.03272-21.4 FIG S4 Analysis of C. albicans behavior after heated EV exposure. (A) The abundance of morphotypes was determined in percentage values in C. albicans underwent heated EVs in comparison with NO EVs condition. (B) RT-qPCR to assess the *HWP1* transcript levels at the 1-h time point. Relative expression was assessed using the NO EVs condition as a reference sample, and the *RPP2B* and *TDH3* genes as reference of normalization. Significantly different values are indicated by asterisks, as determined using ANOVA followed by Tukey’s *post hoc* test (*, *P* < 0.05). Download FIG S4, EPS file, 0.9 MB. Copyright © 2022 Bitencourt et al. 2022 Bitencourt et al. https://creativecommons.org/licenses/by/4.0/ This content is distributed under the terms of the Creative Commons Attribution 4.0 International license.

**FIG 5:**
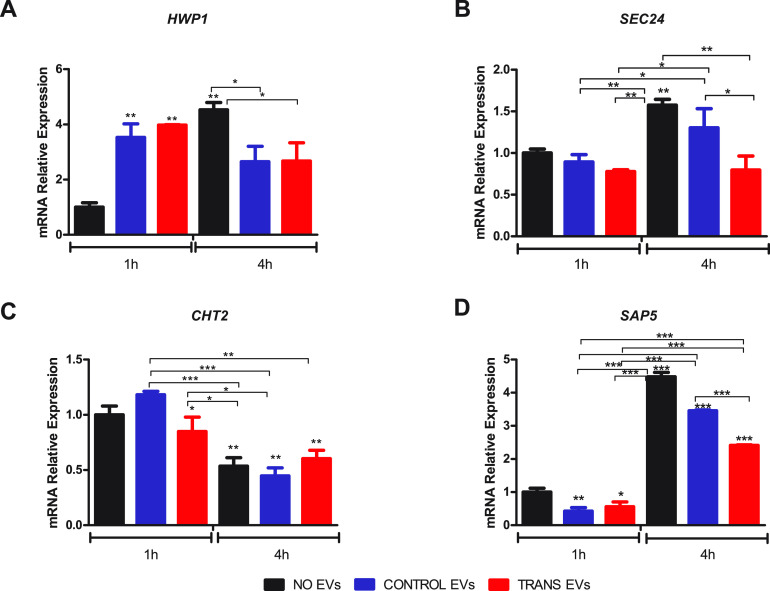
EVs prompted a boost in yeast-to-hypha transition gene expression response. The EVs uptake was carried out with EVs (5 × 10^8^/ml) and C. albicans in cellular density adjusted to an OD_600_ of 0.100 to 0.130. RT-qPCR evaluated a set of C. albicans genes after EVs uptake during 1 h and 4 h of growth on transition cultures. *HWP1* (A), *SEC24* (B), *CHT2* (C), and SAP*5* (D). The relative expression was assessed using NO EVs condition at the 1-h time point as the reference sample after normalization with the *RPP2B* and *TDH3* genes. Significantly different values are indicated by asterisks, determined using ANOVA followed by Tukey’s *post hoc* test (*, *P* < 0.05; **, *P*< 0.01; ***, *P* < 0.001).

EVs uptake caused a rapid response to hypha-inducing stimuli, with a more significant upregulation of the *HWP1* gene at an earlier incubation time point (1 h) followed by a decay in its transcript levels later (4 h), which was observed for both uptake conditions, CONTROL EVs and TRANS EVs (Fig. 5A). The transition panel (Fig. S3) suggested a fluctuation in *HWP1* modulation, with a higher increase in the transcript levels after 2 h, showing an approximately 50-fold difference. In addition, the EVs from control and transition cultures promoted a repression of the *SEC24* gene after 4 h, which was more pronounced by TRANS EVs treatment (Fig. 5B). The transition panel demonstrated its downregulation, particularly in delayed incubation time points, in which hyphae were more commonly found. In addition, the *CHT2* gene was repressed after EVs uptake from transition cultures after 1 h, and no change in its transcription levels was observed after 4 h compared with the NO EVs condition at the same time point (Fig. 5C). A previous study showed that the downregulation of this *CHT2* gene is a remarkable feature in hyphae (43). Therefore, our data suggest a boost in hypha induction after EVs stimulation. Curiously, *SAP5*, which is mainly upregulated in cells with hyphal morphology, presented a decrease in transcription levels under EVs uptake conditions (Fig. 5D). We also investigated the effects of heated EVs on the regulation of the *HWP1* gene and observed a subtle induction triggered by CONTROL EVs (heated), with an increase in transcript levels of about 1.34-fold. No differences in gene modulation were induced by TRANS EVs (heated) compared with NO EVs (Fig. S4).

## DISCUSSION

Intercellular communication occurs through contact with cells or by the secretion of molecules such as quorum-sensing (QS) effectors. A recent study demonstrated that EVs enriched with Fks1 and Chs3 from Saccharomyces cerevisiae rescued the yeast cells from cell wall disturbance (44), which suggested the possibility that EVs function in fungal intraspecies communication.

Here, we evaluated whether EVs are capable of mediating intraspecies communication in fungi by applying three approaches in three different fungal pathogens. We used uptake times of 1 and 2 h, which were sufficient to allow the incorporation of radioactive EVs or at least to promote their aggregation with fungal cells, as mentioned above (Fig. 1). It is expected that subtle differences in EVs uptake could be responsible for altering and regulating gene expression, because fungi have the refined ability to sense any change in the environment and adjust their cellular machinery in response to these changes.

Our study demonstrated a role for EVs as stress message effectors in the fungal agents *P. brasiliensis* and A. fumigatus. In the *P. brasiliensis* model, the genes belonging to the UPR pathway were studied after cultures underwent EVs uptake (EVs were obtained from previous tunicamycin-exposed cultures) compared to cells directly exposed to tunicamycin. The UPR pathway is switched on to reestablish endoplasmic reticulum homeostasis by heightening the folding capability and controlling misfolded protein disposal (45). In fungi, this pathway comprises the ER-transmembrane sensor Ire1/IreA (Ser/Thr kinase) and transcription factor Hac1/HacA (46). Our experiments demonstrated the upregulation of both genes after TM EVs uptake, suggesting that EVs function in fungal communication.

In the A. fumigatus model, EVs obtained from a regular or an irradiated culture triggered a stress response in fresh A. fumigatus cultures, as evidenced by colony reduction formation and upregulation of a stress response gene, the *mpkC* gene. We hypothesize that fungitoxic compounds in EVs from A. fumigatus are responsible for these effects. Previously, the production of antifungal compounds by filamentous fungi such as A. fumigatus was reported (47). However, the characterization and understanding of the processes mediated by EVs in A. fumigatus have been poorly explored. Recently, a study showed the production and cargo description of EVs from A. fumigatus in which approximately 60 proteins were identified with potential roles in immunomodulation and pathogenicity (19).

In this study, the uptake of EVs from cultures exposed to UV light prompted the most pronounced upregulation of the *mpkC* gene. It also promoted a remarkable increase in transcript levels of the *akuA* gene. *akuA* is involved in DNA repair (39). UV exposure causes DNA damage through mutation and pyrimidine dimers and induces oxidative stress status (48, 49). A previous study demonstrated the involvement of *akuA* in cellular protection against UV radiation exposure, showing that *akuA* deletion was responsible for a significant reduction in survival rates of the mutant strain compared to the A. fumigatus wild type (50). Moreover, we revealed that the cultures that underwent EVs uptake from A. fumigatus showed a trend of downregulating an encoding gene of cyclin-dependent kinase, NimA, involved in cell cycle transition (51).

In C. albicans, we assessed the potential of EVs to favor hyphal formation. It is widely accepted that dimorphism in C. albicans is important for virulence (52). The yeast-to-hypha transition is a tightly regulated process of sensing and responding to environmental cues (53). Hypha formation is mainly associated with invasion and adaptive responses during fungus-host interactions (54). Recently, single-cell transcriptome sequencing data showed the dynamic regulation of transition genes during macrophage infection with C. albicans and also highlighted the occurrence of bimodal modulation in genes related to hypha formation and cell wall remodeling concomitantly with differences in immunomodulation responses (55).

In our data, we detected a new response characterized by cell clumping after TRANS EVs uptake in which the EVs were obtained from C. albicans hypha-inducing cultures. Cell clumping effects probably reflect changes in surface hydrophobicity. This feature may result from cell surface changes activated in the regulation of hyphal expansion (56, 57). Furthermore, the gene modulation profile assessed after EVs uptake also showed a boost in yeast-to-hypha transition response, mainly prompted after TRANS EVs uptake, which is reinforced by the presence of long hyphae in this treatment (Fig. 4D). Hyphal growth is associated with a hypha-specific transcriptional program that paves the way toward the expression of genes related to virulence traits such as adhesion (ALS3 and HWP1), invasion (ALS3), oxidative stress response (SOD5), proteolytic activity (SAPs), and others (54). In a previous high-throughput study, a set of genes showed differential modulation after transition stimuli, including the genes *SAP5*, *HWP1*, *SEC24*, and *CHT2* (42). Furthermore, hyphal development relies on a complex network of transduction signals that respond to environmental cues and activate the molecular processes involved in driving the high-polarization growth of hyphal forms (58, 59).

Aspects of cell cycle organization are also unique in hyphae compared to pseudohyphae and yeasts. Hyphal forms showed different branching patterns, regulation of the cell cycle, and polarization (59). In addition, a phenomenon known as QS governs responses related to hyphal formation and control of cell density (60, 61). Many QS molecules might also be found within fungal EVs; however, it is also possible to speculate a cross-regulation between small RNA content in EVs and QS phenomena in fungi. A recent study showed the involvement of QS molecules such as farnesol derivatives and medium-chain fatty acids in EVs obtained from yeast culture as potential regulators of cellular proliferation and yeast-to-hypha development (32). The EVs from C. albicans reduced the hypha and biofilm formation and the impact of C. albicans EVs on invasive hypha development and virulence, as shown in a Galleria mellonella model (32). Moreover, that study was conducted exploring EVs obtained from yeast cultures, and it is reasonable that the cargo and the message within these EVs contributing to cells remain in this form or just evolve toward pseudohyphal morphology, as inferred from our data.

Current knowledge regarding the differences in cell cycle regulation in hyphal development associated with our data prompted the investigation of cellular proliferation after EVs uptake. We demonstrated that the conditions that underwent EV uptake presented an increase in mitochondrial activity, suggesting a function of EVs in heightening cellular metabolism and/or proliferation. EVs were previously shown to enhance cellular proliferation in C. albicans (32) as well as in C. neoformans within macrophages. The high activation of the C. neoformans proliferation rate was named the division of labor mechanism (11). It was ascribed to an important virulence trait transferred from an outbreak strain to a nonoutbreak strain (11).

Collectively, our data pave new avenues for EV functions in fungal intraspecies communication, supporting the role of EVs as potent message effectors regulating pathophysiological mechanisms. Although we recognize the need to develop our knowledge about the complex cellular communication circuit switched on by EVs, and regardless of how it will be addressed in the future, this study sheds new light on the role of fungal EVs in intraspecies cellular communication.

## MATERIALS AND METHODS

### Fungal strains and growth conditions.

Paracoccidioides brasiliensis strain 18 (Pb18) was cultivated as previously described (62). The Pb18 yeast form was maintained in Fava Netto semisolid medium and incubated at 36°C. Yeast growth was determined by inoculating cultures in liquid YPD medium (2% peptone, 1% yeast extract, and 2% glucose) at 36°C on a rotary shaker at 100 rpm for 72 h. Tunicamycin (TM) was used to induce ER stress. TM (15 μg/ml) was prepared in 20 mM NaOH, and it was added to yeast cultures in liquid YPD for 5 days at 36°C on a rotary shaker at 100 rpm to obtain EVs derived from ER-stressed fungi. The same fungal growth was seen without TM to obtain Pb18 CONTROL EVs.

The Aspergillus fumigatus strain CEA17 used in this study was grown in a complete agar malt medium (YAG medium supplemented with malt extract: 2% [wt/vol] glucose, 0.2% [wt/vol] yeast extract, 2% [wt/vol] malt extract, 2% [wt/vol] agar, and 0.1% [vol/vol] trace elements) for 7 days at 37°C. Conidial suspensions were obtained from 7-day-old plates with sterilized PBS, recovered by centrifugation, and filtered through sterile Miracloth (Millipore, Billerica, MA, USA). Conidial concentration was determined using a Neubauer chamber. Approximately 1 × 10^3^ conidia were directly inoculated into complete YAG medium or the conidial suspension was first exposed to UV light for 60 s and then inoculated into a complete YAG medium. The cultures were incubated for 2 days at 37°C. These plates were used for the following experiments: EV isolation and communication assay.

Candida albicans strain ATTC-64548 was grown at 30°C in Sabouraud medium (Oxoid, Basingstoke, United Kingdom) for 72 h. One fresh colony was inoculated into 10 ml of yeast extract-peptone-dextrose medium (YPD pH 6.3) and cultured overnight at 30°C with shaking (150 rpm). The overnight cultures of C. albicans then were diluted to an optical density at 600 nm (OD_600_) range of 0.100 to 0.130 with YPD medium (pH 6.3) or YPD medium (pH 7.4) and grown at 30°C or 37°C, respectively. The cultures grown on YPD at pH 6.3 represent the control cultures in which no stimuli for hypha differentiation were offered, whereas cultures grown on YPD at pH 7.4 represent the transition cultures in which a stimulus conferred by pH (7.4) and temperature (37°C) were offered to prompt hypha differentiation. After that, the cultures were used to perform the EVs isolation experiment, the gene expression profile of yeast to hyphae, and the communication assay.

### EVs isolation.

EVs from Pb18 yeast cultivated in the presence or absence of TM were isolated as previously described (63). Yeast cultures of Pb18 growth in YPD medium were depleted from cell pellets by serial centrifugation at 5,000 × *g* for 15 min and 15,000 × *g* for 30 min at 4°C. The supernatants, containing EVs, were concentrated and filtered using Amicon systems (Millipore, Billerica, MA, USA) with a 100-kDa-cutoff membrane. The concentrated material was centrifuged again at 15,000 × *g* for 30 min at 4°C, and the supernatants were centrifuged at 100,000 × *g* for 1 h at 4°C to collect vesicles. The EVs pellets were resuspended in PBS for nanoparticle-tracking analysis (NTA) and experiments. EVs obtained from TM-treated Pb18 were termed TM EVs, whereas EVs obtained from nontreated cultures were termed Pb EVs.

Regarding the isolation of A. fumigatus EVs, the procedures were performed as previously described (19, 41), with slight modifications. Two-day-old plates obtained for a regular culture or a UV-exposed culture were used to isolate EVs. UV light exposure cultures were obtained after conidial irradiation. Approximately 10^4^ conidia/ml were irradiated with a UV germicidal light (G1578 UV lamp) at 16-cm distance for 60 s with constant shaking, and then 100 μl of this irradiated suspension was plated on YAG medium, yielding about 10% of the CFU compared to nonirradiated cultures. The cells were washed twice with 3 ml of sterile PBS, recovered from the dish plates with inoculation loops, and then transferred to centrifuge tubes. Thereafter, the cell suspension was filtered through sterile Miracloth (Millipore, Billerica, MA, USA), and then a sequential centrifugation and supernatant concentration in the Amicon system was performed. The EVs obtained from UV light exposure were named UV EVs, whereas the EVs obtained from regular cultures were called control EVs.

Isolation of C. albicans EVs was performed as described previously, with slight modifications (9, 44). The concentration of the C. albicans cultures was adjusted as mentioned above, and the preinocula were added into 300 ml of YPD (pH 6.3) or YPD (pH 7.4) and incubated for 4 h at 30°C or 37°C, with shaking (100 rpm). For EVs isolation, the cells and debris were removed by sequential centrifugation at 4,000 × *g* for 15 min and 15,000 × *g* for 15 min. Supernatants were concentrated using an Amicon ultraconcentration system (cutoff, 100 kDa; Millipore, Billerica, MA, USA). The resulting concentrated supernatant was ultracentrifuged at 100,000 × *g* for 1 h at 4°C. Pellets (EV-containing) were collected and resuspended in ultrapure water (Sigma-Aldrich, St. Louis, MO, USA) supplemented with protease inhibitor cocktail (10×; Sigma) (0.2%, vol/vol) to prevent cargo degradation, and then the EVs were stored at −80°C. Taking into account the culture conditions used to promote yeast-to-hypha transition (YPD medium at pH 7.4, incubated at 37°C), an additional step was included in which the cultures were first filtered using sterile Miracloth (Millipore, Billerica, MA, USA) and then subjected to differential centrifugation. EVs obtained after C. albicans grown on YPD at pH 6.3 at 30°C were named CONTROL EVs, while EVs obtained from C. albicans grown on YPD at pH 7.4 and incubated at 37°C were named TRANS EVs.

### NTA.

To determine the size distribution and quantification of EVs isolated from C. albicans cultures in two different stages, a yeast culture condition (CONTROL EVs) and a yeast-to-hypha culture condition (TRANS EVs), NTAs were performed. We also performed the NTA to measure and characterize the size distribution of EVs isolated from regular and UV-exposed A. fumigatus cultures. We obtained the profiles of *P. brasiliensis* EVs as previously described (35). NTA was performed using a Nanosight NS300 appliance (Malvern Instruments, Malvern, UK) with NTA 3.0. The parameters were set as recommended by the manufacturer’s manual. The camera level was increased to a level of >14, in which all particles were distinctly visible, and the threshold was determined to capture as many particles as possible, within an ideal range of 20 to 100 particles per frame. The NTA is an optical dispersion technique employed to measure the size distribution of particles in a solution at the nanometer scale (64).

### Determination of EVs uptake by fungal cells.

To obtain radiolabeled EVs, each one of the fungal species (*P. brasiliensis*, A. fumigatus, and C. albicans) was pulsed with [1-^14^C] palmitic acid for 72 h before EVs isolation (65). The culture medium used for each species and EVs isolation process were described above. Radiolabeled EVs from the same species (10^9^/ml) were added to the corresponding yeast-phase fungi (10^7^/ml) and incubated for 0, 1, 6, 12, and 24 h at 37°C in 9.5% CO_2_. After incubation, culture supernatants containing EVs that were not taken up were removed, fungal cells were washed thrice with PBS and lysed in 200 μl of 25 mM deoxycholate, and the resultant material was collected for scintillation counting.

### Gene expression profile of C. albicans in yeast-to-hypha transition stage.

The C. albicans inoculum was prepared, and its concentration was adjusted as previously described. It was added to 5 ml of YPD (pH 6.3) and incubated for 0.5, 2, and 4 h at 30°C (yeast culture condition). Thereafter, the cultures were centrifuged at 4,000 × *g* for 10 min at 4°C. The resulting pellet was stored at −80°C until RNA extraction. The C. albicans inoculum was also added to 5 ml of YPD (pH 7.4) and incubated for 0.5, 2, and 4 h at 37°C (hypha-yeast culture condition). The pellet obtained from these cultures was stored at −80°C until RNA extraction. We used the resulting material to create a panel that reflects the expression profiles of *HWP1*, S*EC24*, *SAP5*, and *CHT2* genes during the C. albicans transition stage.

### Communication assay mediated by EVs.

**(i) Effect of EVs on *P. brasiliensis* endoplasmic reticulum stress.** The *P. brasiliensis* yeast cells were treated with TM as previously described (36). The EVs from TM-treated and untreated yeast cultures were isolated, and the uptake of EVs (4 × 10^9^/ml) with *P. brasiliensis* yeast cells (10^5^/ml) was performed for 2 h at 37°C in brain heart infusion medium (Sigma). RNA isolation, cDNA synthesis, and quantitative PCR (qPCR) assays were performed as described previously (36).

**(ii) Effects of EVs on A. fumigatus UV stress.** The assay was performed with 5 ml of A. fumigatus conidial suspension (10^4^ conidia/ml). The conidial suspension was incubated with 5 × 10^8^ EVs/ml obtained from A. fumigatus regular cultures or UV light-exposed cultures. Additionally, the EVs from both cultures were incubated at 90°C for 15 min, and these heated EVs were also added with fungal conidia. Uptake was performed for 1 h at 37°C in PBS with shaking (100 rpm). Therefore, the fungal cells were recovered by centrifugation at 4,000 × *g* for 10 min at room temperature, and the cultures were resuspended in 5 ml of PBS. Subsequently, approximately 100 μl of conidia was plated on the YAG medium. After incubation at 37°C in the dark for 2 days, the CFU were counted. CFU from conidia without EVs uptake were counted as controls. The colonies were stored at −80°C until RNA extraction.

**(iii) Effects of EVs on C. albicans dimorphism.** About 5 × 10^8^ EVs obtained from C. albicans yeast cultures (CONTROL EVs) or C. albicans yeast-to-hypha transition cultures (TRANS EVs) were added into a fresh culture of C. albicans with the cell density adjusted to an OD_600_ of 0.10 to 0.130, as previously mentioned, in 5 ml of YPD (pH 6.3 for 1 h at 30°C), with shaking (100 rpm). Additionally, the EVs obtained from both C. albicans cultures were incubated at 90°C for 15 min, and these heated EVs were also added into the fresh yeast culture, as mentioned above. The yeast cells were then recovered by centrifugation. The pellets were resuspended in 5 ml of YPD (pH 7.4) and incubated for 1 h and 4 h at 37°C under shaking (100 rpm). After incubation, the cultures were centrifuged at 4,000 × *g* for 10 min at 4°C, and the pellet was stored at −80°C until RNA extraction. Similar conditions were employed to analyze cellular proliferation through the 2,3-bis-(2-methoxy-4-nitro-5-sulfophenyl)-5-[carbonyl (phenylamino)]-2H-tetrazolium hydroxide (XTT) reduction assay. The XTT assay was performed as previously described (66), with slight modifications. After the uptake of EVs in C. albicans cultures, the yeast pellets were recovered through centrifugation at 4,000 × *g* for 10 min at room temperature, and the pellets were resuspended in YPD (pH 7.4) and incubated for 2 h at 37°C. The medium then was removed, and the pellet was resuspended in XTT solution (1 mg/ml in PBS) and menadione (1 mM in acetone) and incubated for 3 h with gentle shaking. The activity of the yeast mitochondrial dehydrogenase reduces the tetrazolium salt XTT to formazan salts, which results in a colorimetric change that might correlate with cell viability. Colorimetric changes were measured using an enzyme-linked immunosorbent assay microplate reader (Multiskan FC; Thermo Scientific) at 450 nm. Additionally, we monitored the changes in yeast-to-hypha transition in C. albicans cultures after EVs uptake by microscopy analysis. Images were obtained using a Zeiss Observer Z.1 microscope with AxioVision SE64 software after 1-h, 2-h, and 4-h time points. As controls, cultures that had not undergone the EVs uptake process were analyzed.

### RNA extraction and RT-qPCR.

The C. albicans cells were treated with lysis solution (20 mg/ml lysozyme, 0.7 M KCl, and 1 M MgSO_4_, pH 6.8) for 1 h with shaking (100 rpm). Next, the supernatant was removed by centrifugation at 1,000 × *g* for 10 min, and total RNA was extracted using the Illustra RNAspin Mini RNA isolation kit (GE Healthcare) by following the manufacturer’s instructions. The A. fumigatus mycelia were lysed by mechanical pulverization with a pestle and mortar in liquid nitrogen, and total RNA extraction was performed using an Illustra RNAspin mini RNA isolation kit (GE Healthcare). *P. brasiliensis* cells were also lysed with the aid of a small mortar and pestle in liquid nitrogen before total RNA was isolated using TRIzol reagent (Life Technologies, Carlsbad, CA, USA), as described previously (36). RNA concentration and quality were estimated using a nanophotometer (Implem). The RNA was pretreated with DNase (Sigma). cDNA synthesis was performed using a high-capacity cDNA reverse transcription kit (Applied Biosystems) by following the manufacturer’s instructions. Real-time qPCR (RT-qPCR) was conducted as described previously (67). The qPCR experiments were performed with SYBR green master mix (Applied Biosystems) in the Step One Plus platform. Primer sequences were retrieved from the IDT DNA primer quest tool (www.idtdna.com/primerquest/Home/Index), and the oligonucleotide sequences are listed in Table 1. The algorithm used for gene expression analysis was the relative quantification 2^−ΔΔCT^ method (68), and graphs were generated using GraphPad Prism v.5 software (GraphPad).

**TABLE 1:** Primers used in RT-qPCR

Gene ID	Sequence (5′–3′)	Concn (nM)	Efficiency (%)
RPP2B	FWD, TGGTGTTGAAGCCGAAGAA; REV, CGGATGGGACAGAAGCTAAT	100	95.18
TDH3	FWD, CACGGTAGATACAAGGGTGAAG; REV, GGAATGTTAGCTGGGTCTCTTT	150	108.6
HWP1	FWD, GCCTGATGACAATCCTCCTATT; REV, GAGTAGTAGCTGGAGTTGTTGG	400	97.75
CHT2	FWD, TGGTGGTGTTGGTGACTATG; REV, CAGCGTCATCAAATGGTCTTTC	300	92.49
SAP5	FWD, GCGAAGCTACCGAGTTTGAT; REV, GCTTCAGCAGAGTTAAGGTAGAG	500	100.53
SEC24	FWD, GAGCTGGATATGGCGGATATG; REV, GTGGAAGGTCCCTTGATAAGTC	300	99.62
18S	FWD, AAGTGCGCGGCAATAACA; REV, CTCGGCCAAGGTGATGTACT	100	89.34
*Btub* ^*a*^	FWD, TTCCCAACAACATCCAGACC; REV, CGACGGAACATAGCAGTGAA	70	119
*akuA*	FWD, GCTCCTGTGTACCTGAAAGATG; REV, GGGACCGACCGAGAATTTATG	70	100.74
*mpkC*	FWD, TTCCGAGGTCCTTGACTATCT; REV, GTTCAAGAGCACTCGGATCAA	100	105.13
*nimA*	FWD, TCAGCGGCAAGCAAGAATA; REV, TGAGGGAAGATCGGGTATATCA	100	92.65
*HACA*	FWD, GATTCACCCACTCTTGTCCC; REV, GAATCTGTGAGGTCCAAGTCC	100	97.38
*IRE1*	FWD, CACAATTTACAGGAGCTTGCG; REV, GAACCCTTGTCTCGTCTAACTC	100	95.43
Α-tubulin	FWD, CGGCTAATGGAAAATACATGGC; REV, GTCTTGGCCTTGAGAGATGCAA	100	93.12

a See reference 69.

### Statistical analyses.

The results are presented as mean values from independent experiments ± standard deviations. Significant differences were determined by one-way analysis of variance (ANOVA) followed by Tukey’s *post hoc* tests or unpaired *t* test, using GraphPad Prism 5 software.
